# Multifaceted role of SARS-CoV-2 structural proteins in lung injury

**DOI:** 10.3389/fimmu.2024.1332440

**Published:** 2024-02-05

**Authors:** Guoping Zheng, Guanguan Qiu, Huifeng Qian, Qiang Shu, Jianguo Xu

**Affiliations:** ^1^Shaoxing Second Hospital, Shaoxing, Zhejiang, China; ^2^The Children’s Hospital of Zhejiang University School of Medicine and National Clinical Research Center for Child Health, Hangzhou, Zhejiang, China

**Keywords:** SARS-CoV-2, COVID-19, acute respiratory distress syndrome (ARDS), structural proteins, acute lung injury

## Abstract

Severe acute respiratory syndrome coronavirus 2 (SARS-CoV-2) is the third human coronavirus to cause acute respiratory distress syndrome (ARDS) and contains four structural proteins: spike, envelope, membrane, and nucleocapsid. An increasing number of studies have demonstrated that all four structural proteins of SARS-CoV-2 are capable of causing lung injury, even without the presence of intact virus. Therefore, the topic of SARS-CoV-2 structural protein-evoked lung injury warrants more attention. In the current article, we first synopsize the structural features of SARS-CoV-2 structural proteins. Second, we discuss the mechanisms for structural protein-induced inflammatory responses *in vitro*. Finally, we list the findings that indicate structural proteins themselves are toxic and sufficient to induce lung injury *in vivo*. Recognizing mechanisms of lung injury triggered by SARS-CoV-2 structural proteins may facilitate the development of targeted modalities in treating COVID-19.

## Introduction

The genome of SARS-CoV-2 contains a single-stranded positive RNA approximately 30 kb in length (1). It encodes 4 structural proteins [spike (S), envelope (E), membrane (M), and nucleocapsid (N)], 9 accessory proteins (open reading frames 3a, 3b, 6, 7a, 7b, 8, 9b, 9c, and 10), and 16 non-structural proteins (NSP 1-16) (1). All four structural proteins are required to complete an infectious event, which includes entering host cells, replication of the viral genome, packaging, assembly, trafficking, and release of virus particles (2). The S protein mediates viral attachment to the target cells via association with angiotensin-converting enzyme 2 (ACE2). S protein is then cleaved by proteases to cause fusion of viral and cellular membranes. Following cellular entry, the viral RNA is replicated, translated, and packaged, resulting in exocytosis of virions and activation of host immune response (3).

COVID-19 initially presents with upper and lower respiratory tract manifestations and later progresses to systemic diseases such as gastrointestinal diseases, myocardial inflammation, and acute respiratory distress syndrome (ARDS), which results from severe acute lung injury (4). A global literature survey at the early phase of global pandemic showed that the fatality ratio of COVID-19-associated ARDS (CARDS) was almost 50%. Moreover, the prevalence of CARDS in non-survivors was as high as 90% (5). In the early stage of CARDS development, alveolar macrophages are activated following viral infection and death of alveolar epithelial cells. Activated alveolar macrophages generate inflammatory cytokines and chemokines that recruit immune cells, including but not limited to T cells, monocytes, and neutrophils, to the alveolar space. In the meantime, the recruited cells augment the production of proinflammatory mediators and culminate in a cytokine storm (6). Concurrently, infection of SARS-CoV-2 elicits direct as well as indirect activation of complement pathways (7). Cytokine storm and complement activation disrupt the epithelial-endothelial barrier and drive endothelialitis, causing elevated permeability as well as accumulation of protein-rich fluid in alveolar and interstitial spaces. Lung pathology and chest computed tomography show evidence of pneumonitis (8). Endothelialitis enhances procoagulant activity and represses fibrinolytic activity, resulting in COVID-19-associated coagulopathy (9). About 80% of patients with viral pneumonitis improve with no specific interventions. Patients with old age, hypertension, diabetes, and obesity are at increased risk of exacerbation and development of CARDS 7-10 days after onset of symptoms (3, 10).

The pathogenesis of CARDS is quite complex and remains to be further explored. S protein of SARS-CoV-1 was reported to aggravate acid-stimulated lung injury via binding with ACE2. In addition, the injury was mitigated by an inhibitor for angiotensin II receptor type 1 (11). Intranasal delivery of recombinant SARS-CoV-1 N protein triggered progressive pulmonary edema in mice (12). Many reports have found that structural proteins of SARS-CoV-2 are sufficient to cause acute lung injury independent of viral infection (13, 14). Our group documented that SARS-CoV-2 N protein prompted acute lung injury in mice via binding with receptor for advanced glycation endproducts (RAGE) and activation of nuclear factor kappa B (NF-ĸB) pathway (15, 16). This article discusses the structural characteristics of SARS-CoV-2 structural proteins, structural protein-provoked proinflammatory responses *in vitro*, and structural protein-evoked lung injury.

## Structural insights into SARS-CoV-2 structural proteins

### S protein

S protein mediates cell recognition, membrane fusion, and entry of the SARS-CoV-2 virus. Variants of SARS-CoV-2 with S protein mutations such as Alpha, Beta, Gamma, Delta, and Omicron augment infectivity and immune escape (17). S protein is comprised of two subunits, S1 and S2. The S1 subunit is composed of a N-terminal domain (NTD) and a long C-terminal receptor-binding domain (RBD), which binds with ACE2 in the membrane of host cells (18). The S2 subunit carries a fusion peptide for viral entry, two heptad repeats, a transmembrane domain, and a C-terminal tail (19). ACE2 is highly expressed in type I and type II alveolar epithelial cells. In contrast, ACE2 has low expression in airway epithelial cells, endothelial cells, and macrophages (20, 21). Single-cell transcriptomic analysis revealed that SARS-CoV-2 infected type I and type II alveolar epithelial cells, basal cells, club cells, and alveolar macrophages (22, 23). After the binding of S1 subunit with cell surface ACE2, the S2 subunit is subjected to proteolytic digestion by transmembrane serine protease 2 (TMPRSS2), resulting in virus-plasma membrane fusion (24). SARS-CoV-2 virion can also gain entry to host cells via the endosomal pathway, in which S2 subunit is cleaved by cathepsin L (24). Upon cell entry, the viral genome is replicated and transcribed by RNA-dependent RNA polymerase (RdRp), which is composed of catalytic nsp12 subunit and nsp7-nsp8 cofactors in a replication–transcription complex (RTC) (25). The RTC also includes nsp13 helicase for RNA unwinding, nsp10/nsp14 exonuclease for proofreading to enhance replication fidelity, and nsp10/nsp14/nsp16 methyltransferase for RNA capping (26). The viral replication culminates in an exorbitant inflammatory response, which is accompanied by systemic cytokine storm and complement activation, as well as excessive activation of macrophages in the lung. The cytokine storm and complement activation cause endothelial dysfunction and elevated vascular permeability. Activated macrophages in the lung also release excessive proinflammatory chemokines, resulting in infiltration of neutrophils and monocyte-derived macrophages (27).

### E protein

E protein is the smallest SARS-CoV-2 structural protein and is indispensable in viral assembly, release, and pathogenesis. E protein is highly conserved during evolution, as evidenced by 96% similarity between SARS-CoV-1 and SARS-CoV-2 (28). It contains three domains: a negatively charged N-terminus, an uncharged transmembrane domain, and a C-terminus containing diverse motifs for posttranslational modification (28). A small percentage of E protein is integrated into the virions, while the bulk of the protein is localized at Golgi and endoplasmic reticulum–Golgi intermediate compartment (ERGIC), participating in viral assembly and release (29). A recombinant SARS-CoV-1 virus without the E protein gene replicated at a slower rate and caused milder lung inflammation compared with the recombinant wild-type virus in a hamster model (30). A SARS-CoV-2 variant with a 12 base pair deletion at the E protein gene showed higher S protein levels in viral culture, indicating the deletion may enhance viral replication (31). E protein possesses two unique structural features. First, it can form cation selective channels named viroporins via homo-oligomerization, in which asparagine 15 (N15) and valine 25 (V25) are essential for the function (32). SARS-CoV-2 E protein elevated the pH value in ERGIC and lysosomes via the viroporin activity (33). A SARS-CoV-1 virus with E protein mutation for viroporin activity showed reduced edema and production of proinflammatory cytokines in mouse lung (34). Second, E protein contains a C-terminal motif (DLLV) which mediates the binding with PDZ proteins to disrupt epithelial barrier and promote viral spread (35).

### M protein

M protein is also essential in the assembly and release of SARS-CoV-2 (36). It is comprised of a short NTD, three transmembrane domains, and a C terminal domain (CTD) situated in the interior of virion (37). The assembly of SARS-CoV-2 virion takes place inside of ERGIC. M protein encompasses a trans-Golgi network localization signal and is transported to the ERGIC a little earlier than S and E, indicating its function in originating the assembly of SARS-CoV-2 (38, 39). M protein itself binds weakly with N protein, however the interaction is strongly elevated with the co-presence of the N protein and RNA (40). It associates with E protein on the membrane of virus like particles and mediates virion release. The binding between M and E is enhanced by ubiquitinating M at position K15 (41). M protein as well as E protein induce the intracellular retention of S protein and ensure that SARS-CoV-2 viral particles are assembled (36).

### N protein

SARS-CoV-2 N protein packages the viral genomic RNA to form helical ribonucleoprotein complex encompassed within viral capsid (42). In addition, N protein enhances viral RNA transcription and replication via liquid–liquid phase separation (43). N protein contains a NTD, a linker region with abundance in serine and arginine residues, and a CTD. NTD forms a right‐handed fist shape with a core of β‐sheet as well as a β‐hairpin region, while CTD exhibits as tightly interlocked homodimer with a rectangular slab shape (44). There is a positively charged RNA binding groove on the surface of NTD (45). Both NTD and CTD participate in binding with the RNA genome, while phosphorylation of the linker region impacts RNA binding (46). The linker region is indispensable for anchoring the ribonucleoprotein to the viral membrane (45). There is a phosphorylation-dependent association between N protein at the linker region and human 14-3-3 family proteins, which may regulate nucleocytoplasmic shuttling of N protein (47). Binding between N protein and ubiquitin-like domain 1 of NSP3 of SARS-CoV-2 induces ribonucleoprotein dissociation (48). N protein also triggers humoral and cellular immune response, suggesting the potential benefits of future COVID-19 vaccine formation containing N component (49, 50).

## Structural protein-induced inflammatory responses *in vitro*

### S protein

Both ACE2 shedding and downregulation of ACE2 play a role in S protein-induced inflammatory response. ACE2 is a transmembrane protein and can be cleaved into soluble but enzymatically active ACE2 through a process called shedding. Binding of S protein to ACE2 induced ectodomain shedding of ACE2 by tumor necrosis factor-alpha convertase (ADAM17) for SARS-CoV-1 (51) and membrane-type 1 matrix metalloproteinase for SARS-CoV-2 (52). Soluble ACE2 interacted with S protein to promote receptor-mediated endocytosis of SARS-CoV-2 (53). Inhibition of the generation of soluble ACE2 reduced the production of TNF-α *in vitro* (54). Soluble ACE2 occurred early in COVID-19 patients and was reported as a predictor of disease severity (55). In terms of ACE2 downregulation, Gao et al. reported that S protein downregulated ACE2 via enhancing the degradation mRNA of ACE2 (56). ACE2 countered the effect of angiotensin II (Ang II) by converting Ang II into Ang 1-7. Reduced ACE2 enhanced the activation of Ang II/Ang II type 1 receptor pathway, leading to proinflammation response and vasoconstriction (57). Higher levels of Ang II were detected in COVID-19 patients (56). In contrast, Lu et al. found that S protein downregulated ACE2 expression via clathrin and AP-2-mediated endocytosis. S protein-primed cells presented with a gene expression pattern of activated cytokine signaling (58). Additionally, downregulation of ACE2 induced by S protein was responsible for endothelial dysfunction, resulting in oxidative stress and inflammation (59).

Several studies have demonstrated that S protein has proinflammatory activity *in vitro*. Villacampa et al. reported that S protein triggered the activation of NF-κB and NLR family pyrin domain containing 3 (NLRP3) inflammasome in endothelial and immune cells (60). NLRP3 inflammasome activated caspase-1, which enhanced the generation of proinflammatory and proapoptotic IL-1β and IL-18 (61). Petruk et al. discovered that S protein bound lipopolysaccharide (LPS) with high affinity and induced NF-κB activation and cytokine responses in several cell types (62). Khan et al. found that S protein was recognized by Toll-like receptor 2 (TLR2), which dimerized with TLR1 or TLR6, to trigger the activation of NF-κB and induce proinflammatory cytokines such as IL-6, TNF-α, and IL-1β (63). Umar et al. revealed that production of S protein-induced proinflammatory cytokines was blocked by TLR2 or TLR7 knockdown in macrophages, indicating the involvement of both TLR2 and TLR7 in S protein signaling (64). However, Zhao et al. showed that S protein bound and activated TLR4 (65). Patra et al. demonstrated that S protein promoted Ang II type 1 receptor signaling, which activated MAPK/NF-κB pathway and induced IL-6 release in epithelial cells (66). Barhoumi et al. uncovered that S protein promoted M1 macrophage polarization, leading to apoptosis, production of reactive oxygen species, and elevated proinflammatory cytokines. These effects were partially blocked by an ACE inhibitor (67). Li et al. showed that S protein promoted autophagy via PI3K/AKT/mTOR pathway in ACE2 expressing cells and enhanced inflammation and apoptotic responses (68). In addition, Olajide et al. reported that recombinant S1 protein stimulated the release of proinflammatory cytokines from peripheral blood mononuclear cells through activation of NF-κB, p38 MAPK, and NLRP3 inflammasome (69).

### E protein

Ion-channeling viroporins formed by E protein promoted the activity of NLRP3 inflammasome, which controlled activation and release of IL-1β and IL-18 (70). E protein interacted with PDZ domain 2 of human zona occludens-1 and caused damages to tight junction and epithelial barrier, contributing to virus spread and accumulation of water in the lungs (71). Equilibrium and kinetic analysis indicated that E protein bound with the tight junction-associated PALS1 with high affinity, resulting in epithelial barrier disruption and amplified tissue remodeling (72). E protein induced the dysfunction of the blood-brain barrier and triggered inflammatory response in a blood-brain barrier model (73). In macrophages primed with LPS and stimulated with an analogue of viral double-stranded RNA (poly I:C), E protein elevated NLRP3 inflammasome activation (74). E protein was recognized by TLR2 to trigger the release of inflammatory mediators including TNF-α and IFN-γ (75). Additionally, intracisternal injection of E protein prompted depression-like symptoms and dysosmia via TLR2-dependent neuroinflammation (76). Furthermore, E protein was documented to trigger the production of high-mobility group box 1 (HMGB1), which elicited proinflammatory response via TGF-β1/SMAD2/3 pathway, resulting in renal fibrosis (77).

### M protein

Interferons (IFNs) type I (IFN-α and IFN-β) and type III (IFN-λ) are cytokines with inherent antiviral activity, which impairs viral replication in infected cells (78). Galani et al. reported that reduced production of type I and III IFNs, as evidenced by enhanced proinflammatory responses, were present in peripheral blood mononuclear cells in a group of severe COVID-19 patients (79). M protein was found to modulate type I IFN generation via binding with TANK-binding kinase 1 (TBK1) and enhancing its degradation via ubiquitination. The reduced TBK1 blocked the activation of interferon regulation factor 3 (IRF3), resulting in diminished production of type I IFN (80). Lei et al. documented that SARS-CoV-2 infection triggered overt but delayed IFN-β production, while M protein inhibited virus-induced IFN-β promoter activation (81). Zheng et al. revealed that M protein functioned to reduce the release of IFN-β and IFN-λ via interacting with proteins in RIG-I/MDA-5 signaling, which recognized cytosolic double-stranded viral RNA and mediated the generation of IFNs. M protein bound with RIG-I, MAVS, and TBK1 and subsequently reduced the binding between MAVS and TBK1, resulting in diminished phosphorylation of IRF3 (82). Ren et al. found that M protein induced cell apoptosis via binding with phosphoinositide-dependent protein kinase-1 (PDK1) and blocking the activation of PKB/Akt pathway, while N protein served as a scaffold for the function of M protein (83).

### N protein

Chen et al. discovered that N protein bound with SMAD3, which suppressed the expression of cystic fibrosis transmembrane conductance regulator (CFTR), leading to increased intracellular Cl^−^ concentration in airway epithelial cells. Subsequently, serum/glucocorticoid regulated kinase 1 sensed the elevated Cl^−^ concentration and triggered an inflammatory response (84). Another study showed that SARS-CoV-2 N protein had the most dramatic effect in stimulating antiviral cytokines and proinflammatory chemokines in comparison to the other six N proteins from coronaviruses. N protein promoted endocytosis of nucleic acids, which was enhanced by RANTES and lactate. Moreover, wild-type SARS-CoV-2 N protein prompted more prominent endocytosis of nucleic acid compared with Omicron counterpart (85). Lopez-Munoz et al. discovered that N protein bound to heparan sulfate in cell surface with high affinity. N protein had high affinity to 11 chemokines and impaired chemokine functions, which may facilitate viral replication and transmission (86). Karwaciak et al. found that N protein induced production of IL-6 from human monocytes and macrophages (87), while the effect was blocked by chlorpromazine via impairing MEK/ERK signaling (88). Qian et al. revealed that N protein elevated the expression of proinflammatory TNF-α, IL-1β, and MCP-1 in addition to ICAM-1 and VCAM-1 in endothelial cells. Endothelial cells were activated by the N protein via TLR2/NF-κB and TLR2/MAPK signal pathways (89). Wu et al. showed that viral RNA triggered liquid-liquid phase separation of N protein. As a result, N protein associated with and activated the TAK1 and IKK enzyme complex, which promoted NF-κB activation and inflammatory response (90).

N protein has also been reported to participate in the suppression of innate immune response. Savellini et al. uncovered that N protein bound with TRIM25, an E3 ubiquitin ligase enzyme, and blocked TRIM25-facilitated RIG-I activation and IFN-β production (91). Zheng et al. documented that N protein subdued expression of ISG56, CXCL10, IFN-β, and IFN-λ, induced by poly (I:C). N protein bound with Ras GTPase-activating protein-binding protein 1 (G3BP1) to reduce the formation of antiviral stress granule and block the activation of RIG-1 by double-stranded RNA (92). Another group identified that CTD of N protein was crucial in the liquid-liquid phase separation of N protein. This separation blocked ubiquitination and aggregation of mitochondrial antiviral signaling protein and inhibited innate immunity (93).

## Structural protein-evoked lung injury

### S protein

**Figure 1:**
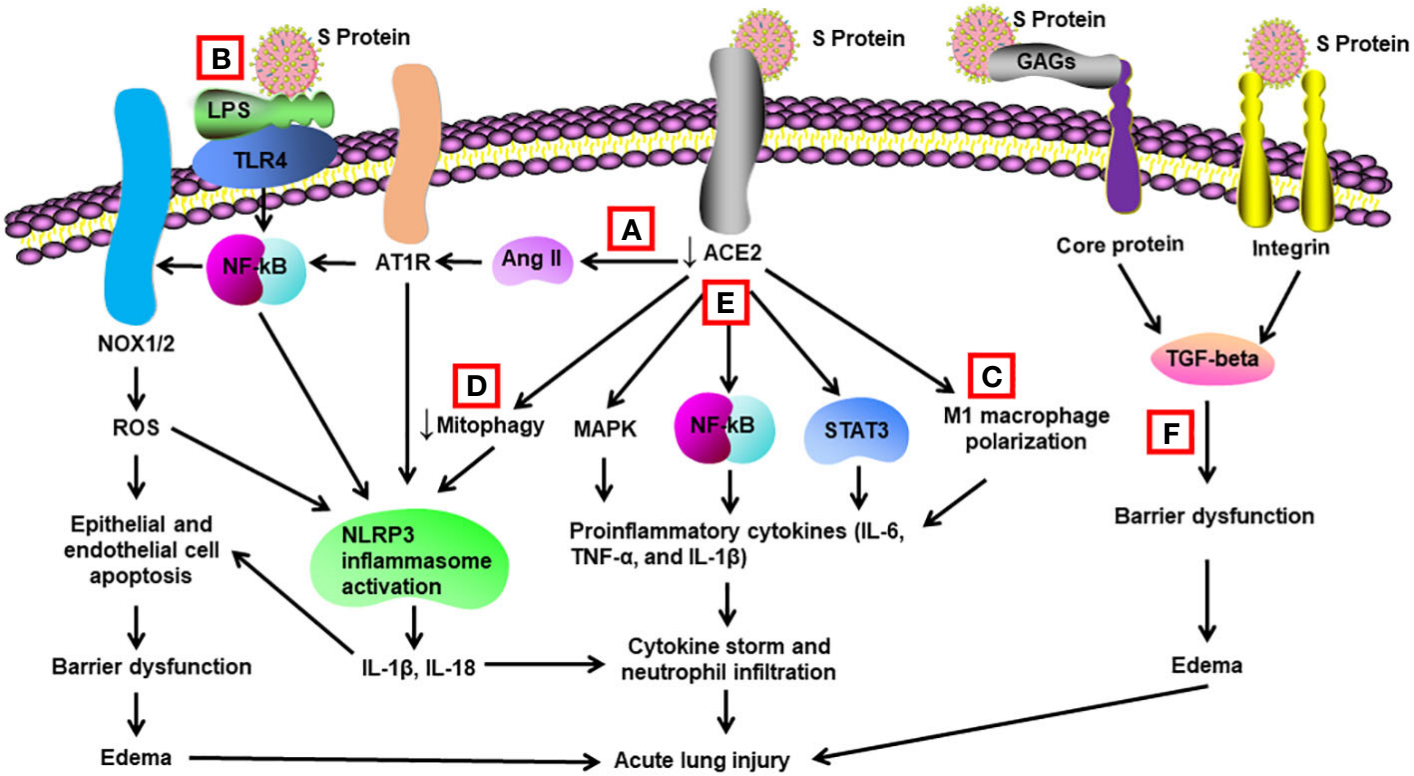
Schematic diagram of mechanisms for S protein-induced lung injury. **(A)** S protein binds with ACE2, leading to ACE2 shedding and downregulation. Reduced ACE2 promotes the activation of Ang II/angiotensin II type 1 receptor (AT1R)/NF-κB/NOX1/2 pathway, leading to generation of reactive oxygen species (ROS). ROS enhances apoptosis of alveolar epithelial cells and endothelial cells, resulting in barrier dysfunction and edema. Activated AT1R also activates NLR family pyrin domain containing 3 (NLRP3) inflammasome in alveolar macrophages and triggers cell apoptosis and cytokine storm. **(B)** S protein activates Toll-like receptors such as TLR4 through binding of lipopolysaccharide (LPS) and enhances NF-κB activity, resulting in activation of NLRP3 inflammasome and cytokine storm. **(C)** S protein promotes M1 polarization of alveolar macrophages through binding to ACE2, which enhances the proinflammatory response. **(D)** S protein reduces mitophagy through binding to ACE2, resulting in activation of NLRP3 inflammasome and cell apoptosis. **(E)** S protein binds to ACE2 and triggers activation of STAT3, MAP kinases (MAPK), and NF-κB via other unidentified mechanisms, leading to cytokine storm and neutrophil infiltration. **(F)** S protein triggers vascular leak and edema independent of ACE2 binding. S protein binds with glycosaminoglycans and integrins, which leads to the activation of TGF-β signaling pathway and barrier dysfunction.

In transgenic mice overexpressing human ACE2, intratracheal instillation of recombinant S1 subunit of S protein elevated cell infiltration and protein concentration in the bronchoalveolar lavage (BAL) at 72 h after exposure. It also upregulated inflammatory cytokines in BAL/serum and induced histological characteristics of acute lung injury. Mechanistically, S protein activated the NF-κB and STAT3 pathways in the lungs (94) (**Table 1**) (**Figure 1**). The same group also reported that S1 subunit of S protein exacerbated lung injury in human ACE2 transgenic mice on an alcohol diet in comparison with mice on a control diet. Concurrently, the S1 subunit activated NF-κB, STAT3, and NLRP3 (95). Cao et al. found that lentivirus expressing S protein targeted type II alveolar cells and M1 macrophages and induced acute lung inflammation in mice at 24 h. Lentiviral S protein also elevated proinflammatory cytokines in the lungs as well as in the RAW264.7 macrophage cell line (96). Using NF-κB reporter mice, Puthia et al. demonstrated that co-administration of S protein and LPS via aerosol synergistically increased NF-κB induction compared with LPS alone. Co-administration of S protein and LPS significantly elevated infiltration of macrophages and neutrophils as well as proinflammatory cytokines in the BAL at 24 h compared with LPS alone. Mice treated with S protein and LPS also had a higher lung injury score in histological analysis. The coadministration model mimicked lung injury observed in COVID-19 (97). In Syrian hamsters, Lei et al. showed that intratracheal administration of pseudovirus expressing S protein induced lung damage with thickened alveolar septa and elevated infiltration of mononuclear cells at day 5. There were decreased levels of phospho-AMP-activated protein kinase, phosphor-ACE2, and ACE2 in the damaged lungs. The alterations in the protein expression were recapitulated in pulmonary arterial endothelial cells infected with pseudovirus expressing S protein (59). Zhang et al. discovered that intraperitoneal injection of recombinant RBD of S protein aggravated LPS-induced acute lung injury in mice at day 3. RBD of S protein bound with ACE2 and downregulated its expression, resulting in an elevation in Ang II. Ang II activated its receptor and downstream NF-κB-NOX1/2 signaling pathway, leading to oxidative stress and redox imbalance as well as proinflammatory response in the lung. In addition, recombinant ACE2 blocked lung injury induced by RBD of S protein (98). In transgenic C57BL/6 mice expressing human ACE2, Liang et al. revealed that intratracheal administration of recombinant RBD of S or S1 protein for 10 days elevated IL-18 mRNA expression in the blood. The treatment augmented infiltration of neutrophils in the lung and lung injury scores. S protein administration also elevated expression of NLRP3-dependent IL-18 in the lung, while IκBα levels were decreased. In addition, S protein increased IL-18 expression via reducing mitophagy and enhancing mitochondrial reactive oxygenation species *in vitro* and *in vivo* (99). Elevated IL-18 levels have been correlated with disease severity and clinical outcomes of COVID-19 patients (99, 102). Satta et al. revealed that intravenous injection of lentivirus expressing S protein increased proinflammatory cytokines in the BAL and macrophages in the lung, which were abolished by liposome-human ACE2 (100). In BALB/c mice expressing human ACE2, Gu et al. found that co-administration of recombinant extracellular domain of S protein and poly (I:C) aggregated lung injury in histology compared with poly (I:C) alone at 24 h. The co-administration also increased neutrophil infiltration and proinflammatory cytokines in the BAL, while S protein or poly (I:C) alone lacked the effect (101). These findings contradicted some other reports cited in this review and indicate that S protein itself does not directly induce significant lung injury but requires coadministration of a pathogen-associated molecular pattern (PAMP). Furthermore, S protein was able to induce lung injury in an ACE2-independent manner. Biering et al. found that S protein triggered endothelial hyperpermeability in cells that do not express ACE2 *in vitro*. Intranasal administration of recombinant S protein triggered vascular leak in the lungs of mice that do not express human ACE2. *In vitro* studies revealed that glycosaminoglycans, integrins, and the TGF-β signaling pathways were all essential for S-mediated barrier dysfunction (13).

**Table 1:** Studies demonstrating the effects of SARS-CoV-2 structural proteins on acute lung injury in animal models.

References	Animal model	Structural protein format	Delivery dose, route, and time of sample collection	Major findings	Mechanisms
Colunga Biancatelli, et al., 2021 (94)	Transgenic C57BL/6 mice expressing human ACE2	Recombinant S1 subunit of S protein	400 µg/kg, intratracheally, 72 h	↑White blood cell infiltration ↑Protein concentrations in BAL ↑Proinflammatory cytokines in BAL and serum ↑Features of lung injury in histology ↑Activation of NF-κB and STAT3	S protein activates NF-κB and STAT3 pathways in the lungs.
Solopov et al., 2022 (95)	Transgenic C57BL/6 mice expressing human ACE2 on alcohol diet	Recombinant S1 subunit of S protein	400 µg/kg, intratracheally, 72 h	↑White blood cell infiltration ↑Proinflammatory cytokines in BAL ↑Features of lung injury in histology ↑Activation of NF-κB, STAT3, and NLRP3 ↑Expression of lung ACE2 in mice with alcohol diet	Alcohol elevates expression of ACE2 in the lung. S protein activates NF-κB, STAT3, and NLRP3 pathways.
Cao et al., 2021 (96)	C57BL/6 mice	Lentivirus expressing S protein	8 × 10^8 viral particles, intravenous injection, 24 h	↑Inflammatory cell infiltration ↑Alveolar wall thickness ↑Proinflammatory cytokines in the lung	S protein induces inflammatory response in lung macrophages.
Puthia et al., 2022 (97)	NF-κB reporter and wild-type C57BL/6 mice	Recombinant S protein	5 μg, aerosol delivery along with LPS, 24 h	↑Macrophages and neutrophils in BAL ↑Proinflammatory cytokines in BAL ↑Lung injury score ↑NF-κB activation	S protein activates NF-κB synergistically with LPS in the lung.
Lei et al., 2021 (59)	Syrian hamsters	Pseudovirus expressing S protein	1 × 10^8 PFU, intratracheally, 5 days	↑Mononuclear cell infiltration ↑Alveolar septal thickness ↓Expression of phopho-ACE2 and ACE2	S protein decreases ACE2 to impair endothelial function.
Zhang et al., 2022 (98)	C57BL/6 mice	Recombinant RBD domain of S protein	5.5 nmol/kg, intraperitoneally, 3 days	↑Lung injury score and edema ↑Cell counts and protein concentrations in BAL ↑Proinflammatory cytokines in BAL ↓Level of ACE2 in the lung	RBD of S protein induces lung injury via reducing ACE2.
Liang et al., 2023 (99)	Transgenic C57BL/6 mice expressing human ACE2	Recombinant RBD of S protein or S1 protein	5 μg daily, intratracheally, 10 days	↑Lung injury score ↑Neutrophil infiltration ↑Proinflammatory IL-18 in the lung ↓Mitophagy	RBD or S1 protein raises IL-18-mediated lung injury via reducing mitophagy.
Satta et al., 2022 (100)	C57BL/6 mice injected with/without liposome-human ACE2	Lentivirus expressing S protein	1 x 10^5 pfu, intravenously, 24 h	↑Proinflammatory cytokines in BAL ↑Macrophages and IL-6 in the lung	Liposome human ACE2 neutralizes S protein-evoked lung inflammation.
Gu et al., 2021 (101)	BALB/c mice expressing human ACE2 via adenovirus infection	Recombinant extracellular domain of S protein	15 μg, intratracheally, 24 h	↑Lung injury in histology ↑Neutrophil infiltration ↑Proinflammatory cytokines in BAL	Extracellular domain of S protein triggers cytokine storm and lung injury.
Biering et al., 2022 (13)	C57BL/6 mice without expressing human ACE2	Recombinant S protein	50 μg, intranasally, 24 h	↑Vascular leak in the lungs	S protein promotes signaling of integrins and TGF-b

ACE2, angiotensin-converting enzyme 2; S protein, spike protein; BAL, bronchoalveolar lavage; NF-κB, nuclear factor kappa B; STAT3, Signal transducer and activator of transcription 3; NLRP3, NLR family pyrin domain containing 3; RBD, receptor binding domain. ↑ indicates increase and ↓ indicates decrease.

S protein also causes injuries in other systems. Nasal inoculation of adenovirus vector expression S1 protein caused olfactory bulb damage and brain inflammation in mice via elevating calcium and decreasing intracerebral acetylcholine production (103). In mice with collagen-induced arthritis, Lee et al. showed that injection of a plasmid encoding S protein exacerbated arthritis via inducing inflammation, autoantibody, and thrombosis (104). Liang et al. documented that intratracheal delivery of S1 and RBD of S protein prompted cardiac dysfunction and elevated expression of IL-18 and NLRP3 in the heart. IL-18 inhibition alleviated S protein-induced cardiac dysfunction (99). Robles et al. discovered that S protein bound with integrin α5β1 in endothelial cells via RGD motif in the RBD domain. The binding activated NF-κB in endothelial cells, resulting in elevated expression of adhesion molecules (VCAM1 and ICAM1) and proinflammatory cytokines, as well as the hyperpermeability of the endothelial cells *in vitro* and *in vivo* (105). Another study observed that S protein elevated VEGF levels in enterocytes via Ras‐Raf‐MEK‐ERK pathway, enhancing vascular hyperpermeability and inflammation. S protein-induced intestinal inflammation was alleviated by both ERK and VEGF inhibitors *in vivo* (106).

### E protein

**Figure 2:**
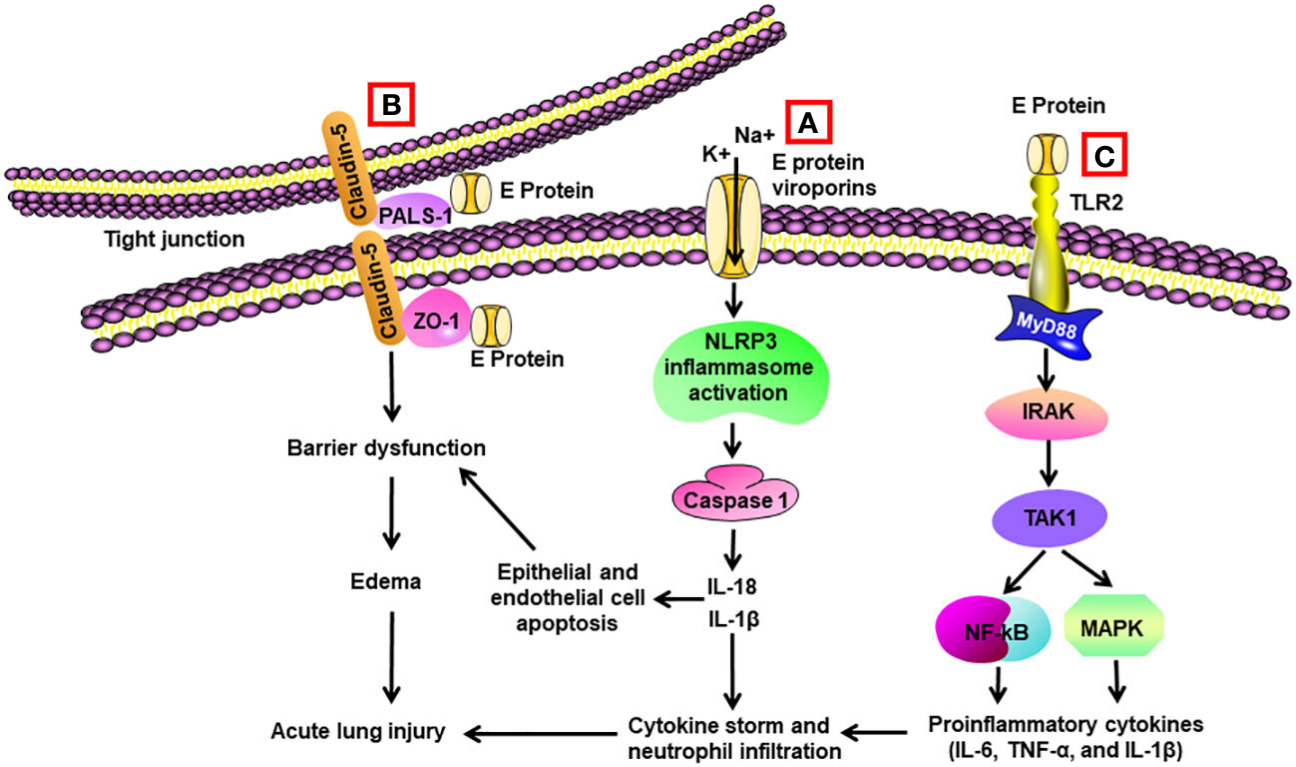
Schematic diagram of mechanisms for E protein-induced lung injury. **(A)** E protein forms ion-channeling viroporins for cations and activates NLRP3 inflammasome in alveolar macrophages, leading to caspase 1 activation and elevated levels of Il-1β and IL-18. IL-1β and IL-18 are the sources of cytokine storm. They also trigger apoptosis of alveolar epithelial cells and endothelial cells, resulting in barrier dysfunction and edema. **(B)** E protein binds with proteins associated with tight junctions such as zona occludens-1 (ZO-1) and PALS-1, leading to barrier dysfunction in epithelial and endothelial cells. **(C)** E protein binds with TLR2 and activates NF-κB and MAPK through activation of the IRAK/TAK1 pathway, resulting in cytokine storm.

Intratracheal administration of recombinant E protein induced the accumulation of inflammatory cells and cell death in the lungs of wild-type (WT) mice at 24 h, which was not present in the TLR2^–/–^ mice. E protein also elevated proinflammatory cytokines in the BAL of WT mice. Blockage of TLR2 reduced SARS-CoV-2 virus-induced mortality and elevation in proinflammatory cytokines in mice (75) (**Table 2**) (**Figure 2**). Another group found that E protein formed pH-sensitive cation channels in an environment of lipid bilayer. Intravenous administration of recombinant E protein produced the hallmarks of acute lung injury with infiltration of inflammatory cells, pulmonary hemorrhage and edema, and interstitial hyperemia in mice at 72 h. There was also an upregulation of proinflammatory cytokines in the serum. In SARS-CoV-2-infected transgenic mice, administration of inhibitors for the channels decreased the viral load, extent of injury, and proinflammatory cytokines in the lungs (107).

**Table 2:** Studies demonstrating the effects of SARS-CoV-2 structural proteins on acute lung injury in animal models.

References	Animal model	Structural protein format	Delivery dose, route, and time of sample collection	Major findings	Mechanisms
Zheng et al., 2021 (75)	WT and TLR2^–/–^ mice	Recombinant E protein	25 μg, intratracheal, 24 h	↑Inflammatory cell infiltration ↑Apoptosis ↑BAL proinflammatory cytokines	E protein binds and activates TLR2.
Xia et al., 2021 (107)	C57BL/6 mice	Recombinant E protein	25 mg/kg, tail vein injection, 6 and 72 h	↑Inflammatory cell infiltration ↑Pulmonary hemorrhage and edema ↑Pulmonary interstitial hyperemia ↑Serum proinflammatory cytokines	E protein forms pH-sensitive cation channels.
Yang et al., 2022 (14)	C57BL/6 mice	Lentivirus expressing M protein	5 × 10^7^ TU/kg, intratracheal, 3 days	↑Pulmonary permeability ↑Apoptosis of lung cells	M protein binds with BOK and promotes mitochondrial apoptosis.
Gao et al., 2022 (108)	C57BL/6 WT and MASP2^−/−^ mice as well as BALB/c mice	Adenovirus expressing N protein	1 × 10^8–9 PFU, tail vein, 24 h after LPS treatment	↑Mortality ↑Lung tissue inflammation	N protein triggers MASP-2-mediated complement activation.
Pan et al., 2021 (109)	WT and NLRP3^−/−^ C57BL/6 mice	Adeno-associated virus expressing N protein	5 × 10^11^ vg, tail veil, 3 weeks	↑Inflammation in histology ↑LPS-induced animal death ↑IL-1β and IL-6	N protein binds and activates NLRP3.
Xia et al., 2021 (15)	WT and TLR4^–/–^ C57BL/6 mice	Recombinant N protein	75 μg, intratracheal, 24 h	↑Protein permeability ↑Total cell count in BAL ↑Neutrophil infiltration ↑Proinflammatory cytokines	N protein induces lung injury via activation of NF-ĸB. N protein induced lung injury is TLR4 independent.
Xia et al., 2023 (16)	WT and RAGE^–/–^ C57BL/6 mice	Recombinant N protein	75 μg, intratracheal, 24 h	↓Lung injury in RAGE^–/–^ mice ↓Lung injury in mice received RAGE antagonist	N protein binds with RAGE and activates RAGE-ERK1/2-NF-ĸB pathway.

WT, wild-type; TLR2, Toll-like receptor 2; BAL, bronchoalveolar lavage; BOK, B cell lymphoma 2 (BCL-2) ovarian killer; MASP-2, MBL-associated serine protease-2; NLRP3, NLR family pyrin domain containing 3; NF-κB, nuclear factor kappa B; RAGE, receptor for advanced glycation endproducts. ↑ indicates increase and ↓ indicates decrease.

### M protein

**Figure 3:**
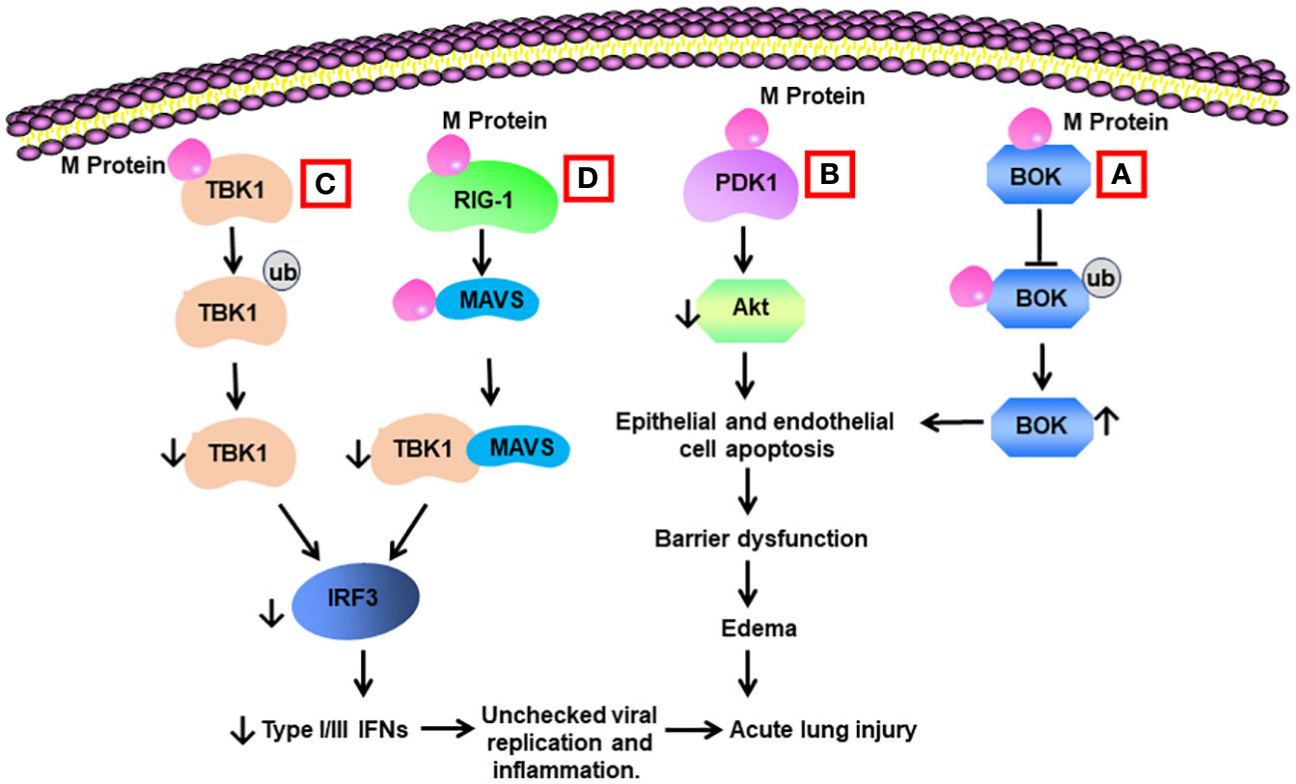
Schematic diagram of mechanisms for M protein-induced lung injury. **(A)** M protein binds with B cell lymphoma 2 (BCL-2) ovarian killer (BOK). The association inhibits the ubiquitination of BOK and increases BOK levels. BOK induces apoptosis of alveolar epithelial cells and endothelial cells, resulting in barrier dysfunction and edema. **(B)** M protein binds with phosphoinositide-dependent protein kinase-1 (PDK1) and downregulates the activity of AKT, leading to cell apoptosis. **(C)** M protein binds with TANK-binding kinase 1 (TBK1) and promotes its degradation via ubiquitination. Reduced levels of TBK1 cause decreased activity of interferon regulation factor 3 (IRF3), leading to low production of type I and type III IFNs. Low type I and type III IFNs contribute to unchecked viral replication and inflammation. **(D)** M protein binds with RIG-1, MAVS, and TBK1 and decreases the phosphorylation of IRF3 via blocking the association between MAVS and TBK1.

Yang et al. reported that M protein bound to BH2 region of B cell lymphoma 2 (BCL-2) ovarian killer (BOK), blocked the ubiquitination of BOK, elevated BOK levels, and promoted mitochondrial apoptosis *in vitro*. Lentiviral expression of M protein elevated pulmonary permeability and prompted apoptosis of lung cells *in vivo*. Knockdown of BOK ameliorated alveolar-capillary permeability and pulmonary edema induced by M protein (14) (**Table 2**) (**Figure 3**).

### N protein

**Figure 4:**
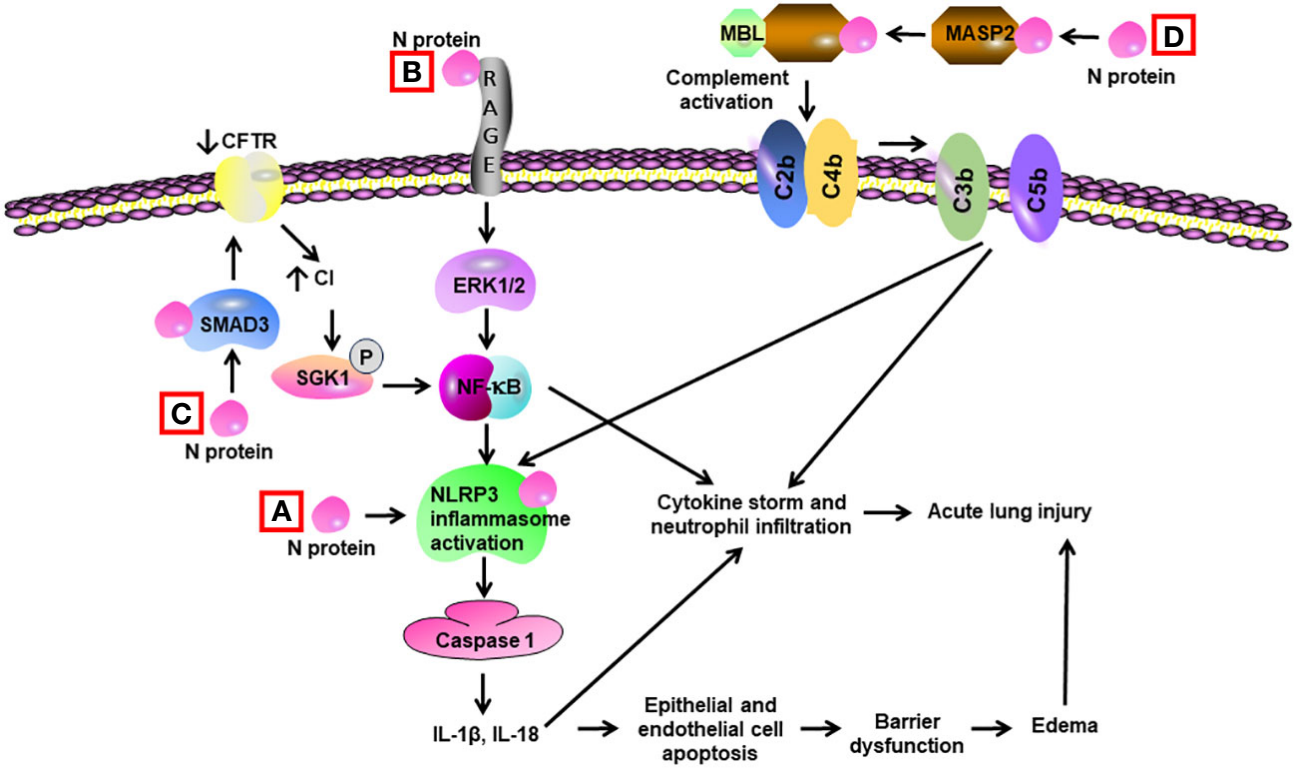
Schematic diagram of mechanisms for N protein-induced lung injury. **(A)** N protein binds and activates NLRP3 inflammasome in alveolar macrophages, leading to activation of caspase 1 and elevated levels of Il-1β and IL-18. These cytokines induce a cytokine storm and neutrophil infiltration. They also trigger apoptosis of alveolar epithelial cells and endothelial cells, resulting in barrier dysfunction and edema. **(B)** N protein binds with RAGE and subsequently activates ERK1/2 in alveolar macrophages. ERK1/2 triggers the activation of NF-κB, leading to cytokine storm. **(C)** N protein binds with SMAD3 and subsequently decreases the expression of cystic fibrosis transmembrane conductance regulator (CFTR), resulting in elevated intracellular Cl^−^ concentration in airway epithelial cells. An increase in concentrations of Cl^−^ causes phosphorylation of serum/glucocorticoid-regulated kinase 1 (SGK1) and activation of NF-κB. **(D)** N protein binds and activates the MBL-associated serine protease-2 (MASP-2), leading to activation of complement cascade via cleavage of C2 and C4 into C3/C5 convertases (C4bC2b). Complement activation leads to cytokine storm and activation of NLRP3 inflammasome.

Gao et al. revealed that N protein bound and activated MBL-associated serine protease-2 (MASP-2), leading to activation of complements 3 and 5b-9. Adenovirus expressing N protein aggravated LPS-induced mortality and lung tissue inflammation in mice. The impact of N protein *in vivo* was blocked by an inhibitor of MASP-2 and antibodies for N protein and MASP-2 (108) (**Table 2**) (**Figure 4**). Pan et al. discovered that N protein bound with NLRP3 to enhance the assembly of NLRP3 inflammasome. Adeno-associated virus expressing N protein induced lung inflammation in histology, aggravated LPS-induced animal death, and elevated the expression of IL-1β and IL-6 in serum as well as in the lung. N protein-evoked lung injury was hindered by inhibitors for caspase-1 and NLRP3 (109). Our group showed that administration of recombinant N protein to C57BL/6 mice prompted acute lung injury, as reflected by increased protein permeability, proinflammatory cytokines, and infiltration of neutrophils in the BAL. N protein also induced M1 macrophage polarization of alveolar macrophages and phosphorylation of NF-ĸB p65 (15). Our group recently revealed that N protein is a ligand for RAGE. N protein triggered proinflammatory response via RAGE-ERK1/2-NF-ĸB pathway. In mice, RAGE knockout and inhibition partially alleviated N-protein-evoked lung injury (16). Wick et al. demonstrated that N protein levels of plasma samples harvested within 72 h of hospital admission were strongly associated with RAGE and correlated with ICU admission as well as mechanical ventilation at 28 days (110). Furthermore, Matthay et al. found that high levels of N protein and RAGE at admission were significantly correlated with the development of severe COVID-19 (111).

## Conclusions

Up to the present, there have been three zoonotic coronaviruses (SARS-CoV-1, MERS-CoV, and SARS-CoV-2) that cause human ARDS. With the ever-increasing intrusion of natural habits, it is foreseeable that novel coronavirus diseases will emerge and spread via the respiratory system. All four structural proteins of SARS-CoV-2 are essential in assembly and release of the virion. The N protein binds to the genomic RNA of SARS-CoV-2, while S is indispensable in viral attachment and entry to target cells. Existing findings have demonstrated that all four structural proteins of SARS-CoV-2 are able to trigger lung injury independent of viral infection. Much work remains to be performed to decipher the molecular mechanisms of structural protein-evoked lung injury and the implications for the injury in humans. Antibody cocktail of structural proteins may represent a new therapeutic tool for treating COVID-19.

## Author contributions

GZ: Conceptualization, Funding acquisition, Writing – original draft, Writing – review & editing. GQ: Writing – original draft, Writing – review & editing. HQ: Writing – original draft, Writing – review & editing. QS: Conceptualization, Funding acquisition, Writing – original draft, Writing – review & editing. JX: Conceptualization, Writing – original draft, Writing – review & editing.
